# Validity and Reliability of the Korean Version of the Utrecht Scale for Evaluation of Rehabilitation-Participation

**DOI:** 10.1155/2017/9452051

**Published:** 2017-01-23

**Authors:** Joo-Hyun Lee, Ji-Hyuk Park, Yeong Jo Kim, Sang Heon Lee, Marcel W. M. Post, Hae Yean Park

**Affiliations:** ^1^Department of Occupational Therapy, Yonsei University, Wonju, Republic of Korea; ^2^Department of Occupational Therapy, Soonchunhyang University, Cheonan, Republic of Korea; ^3^Brain Center Rudolf Magnus and Center of Excellence in Rehabilitation Medicine, University Medical Center Utrecht and De Hoogstraat Rehabilitation, Utrecht, Netherlands

## Abstract

This study investigated the reliability and validity of the Korean version of the Utrecht Scale for Evaluation of Rehabilitation-Participation (K-USER-P) in patients with stroke. Stroke patients participated in this study. The Utrecht Scale for Evaluation of Rehabilitation-Participation was translated from English into Korean. A total of 120 questionnaires involving the K-USER-P were distributed to rehabilitation hospitals and centers by mail. Of those, 100 questionnaires were returned and 67 were included in the final analysis after exclusion of questionnaires with insufficient responses. We analyzed the questionnaires for internal consistency, test-retest reliability, and construct validity. The results indicated that internal consistency coefficients of the frequency, restriction, and satisfaction domains were 0.69, 0.66, and 0.67, respectively. Test-retest reliability was 0.63, 0.45, and 0.71 for the three domains, respectively. Intercorrelations between the SF-12 and the London Handicap Scale were generally moderate to good. The Korean version of the Utrecht Scale for Evaluation of Rehabilitation-Participation can be used as a measure of the participation level of stroke patients in clinical practice and the local community.

## 1. Introduction

Participation is an important outcome of rehabilitation treatment. Particularly in community-based settings and outpatient clinics, the final goal of rehabilitation is to improve the level of activities of daily life participation rather than the level of function [1]. The participation has been described as involvement in actual life situations by International Classification of Functioning, Disability and Health (ICF) framework [2]. For patients with disabilities, increasing this participation in daily livings not only strengthens the well-being of them, but also helps reduce the long-term costs of treatment and support [3]. Thus, in rehabilitation field, there is a growing interest in participation enhancement in daily activities and meaningful life roles and then improvement of functional ability after becoming disabled [4]. A participation measure is therefore needed as an outcome measure to evaluate the effects of rehabilitation interventions.

It is important for therapists to measure objective and subjective participation for patients because participation can explain objective experience such as frequency and duration and subjective expression of patient's values such as satisfaction and importance [5]. Since the introduction of the ICF in 2001, various tools for participation measurements have been developed [6, 7]. However, there is a lack of measures that include both the subjective and objective aspects of participation [1]. Of tools for participation measurement, most tools are objective and normative tools that were used variables such as frequency, duration, and limitations of activities, but not subjective tools [6, 8].

The Utrecht Scale for the Evaluation of Rehabilitation-Participation (USER-P) was developed to measure both objective and subjective participation [9]. Also, the USER-P is a valid and reliable measurement of participation in rehabilitation [1].

Among all neurological disorders, stroke is the leading cause of death and disability in Korea. Stroke survivors experience motor and cognitive impairment or decline such as paralysis, abnormal muscle tone, attention, and memory loss [10]. These stroke-related disabilities can severely restrict participation in meaningful activities of daily living [11]. In other words, patients with stroke are dependent upon others in some of the basic activities of daily living (BADL) such as dressing, transferring to shower, and walking outside as well as the instrument activities of daily living (IADL) such as household tasks [12]. These results can decrease quality of life with patients. So, nowadays goal of intervention and assessment in rehabilitation area aims to promote independence and reintegration into the community of people with stroke [13]. To do this, it is important to measure participation of activities of daily living with stroke patients.

However, participation limitation is not yet widely used as measurement index in the rehabilitation area of Korea [14]. Thus the USER-P can be used to verify effectiveness of intervention and measure the recovery after stroke in Korea.

Therefore, we translated the USER-P into Korean in order to use the tool in our country. The purpose of this study was to test the validity and reliability of the Korean translation of the USER-P.

## 2. Methods

### 2.1. Participants

Study participants were recruited through rehabilitation hospitals and centers in Wonju, Korea. Patients were eligible to participate in the study if (i) they were at least 18 years of age, (ii) they had experienced stroke at least six months prior to the study, (iii) they were able to read Korean, (iv) they had participated in rehabilitation therapy for a period of at least four weeks, and (v) they were enrolled from both inpatient or outpatient settings. Exclusion criteria included patients who (i) have severe aphasia and cognitive impairments confirmed by medical records, (ii) have a fast-progressing medical condition after stroke, and (iii) had not signed an informed consent. Informed consent was obtained from all participants.

### 2.2. Procedure

This study was performed over a period of three months from November 2013 to January 2014. In the first month, the USER-P was translated from English to Korean, and it was back translated into English and compared to the original English USER-P questionnaire by two experienced occupational therapists (bilingual Korea-English speaker). The USER-P Korean version was subsequently developed through review about understanding of contents by a committee of experts (two occupational therapists and two professors) [15]. In the second month, a total of 120 questionnaires involving the USER-P Korean version were distributed to the head or person in charge of rehabilitation hospitals and centers by mail. The questionnaires then were sent to participants by the head in charge of organization. The stroke patients belonging to each organization were requested to complete the questionnaire with instructions. In addition, to analyze test-retest reliability, we distributed another 30 questionnaires to patients 2 weeks after the first distribution.

### 2.3. Instruments

#### 2.3.1. USER-P

USER-P was developed as an instrument to objectively and subjectively measure participation in activities of daily living by people with limited physical functions [1]. The tool consisted of a total of 31 items covering three domains: frequency, restriction, and satisfaction. The frequency domain is comprised of both vocational activities and leisure and social activities. The activities related to vocation consist of paid work, unpaid work, volunteer work, and housekeeping and are measured as the number of hours the respondent spends performing these activities in a typical week. For each item, scores range from 0 (not at all) to 5 (36 hours or more). These items also assess the frequency of activities in the previous four weeks. Each item score ranges from 0 (not possible at all) to 5 (19 times or more).

The restriction domain is comprised of 10 items measuring participation restriction in work, leisure, and social activities as a result of health problems. Each item score ranges from 0 (not possible) to 3 (independent without difficulty). Activities that are not relevant to the subject were rated as “not applicable.”

The satisfaction domain is comprised of nine items measuring satisfaction with work, leisure, and social activities. Items are rated on a scale of 0 (very dissatisfied) to 4 (very satisfied). The total sum of each frequency, restriction, and satisfaction domain is converted into a score ranging from 0 to 100, with higher scores indicating greater participation. The test-retest intraclass correlation coefficient (ICC) of USER-P was 0.65 (0.45–0.79) for the frequency subscale, 0.85 (0.75–0.92) for the restrictions, and 0.84 (0.73–0.91) for the satisfaction. Cronbach's *α* was 0.70 for the frequency domain, 0.91 for the restriction domain, and 0.88 for the satisfaction domain. In addition, this tool had good responsiveness among stroke patients [14]. We used the K-USER-P translated into Korea for reliability and validity analysis.

#### 2.3.2. Other Measures

Two criterion measures, which were SF-12 Korean second version and London Handicap Scale Korean version, were used to study the construct validity of the K-USER-P. These measures can be represented with participation concepts that are being measured such as health status, quality of life, and restrictions. The SF-12 Korean second version is an instrument measuring general health status and quality of life related to health [16]. This instrument is a questionnaire with 12 items, including a physical component summary (PCS) and a mental component summary (MCS). The PCS domain is comprised of physical functioning (PF), role physical (RP), bodily pain (BP), and general health (GH). The MCS domain includes mental health (MH), role emotional (RE), social functioning (SF), and vitality (VT). Each item is scored on a 1–6 scale. The total item scores range from 0 to 100, computed by standard score conversion, and higher scores indicate a better health status. This tool has high reliability, with Cronbach's *α* ranging from 0.81 to 0.84 [17].

The London Handicap Scale (LHS) Korean version is a self-completed questionnaire measuring the effect of chronic disorders on a person's functional ability [16]. It is composed of six items: mobility, physical independence, occupation, social integration, orientation, and the economic self. Each item is scored on a Likert scale of 1 (no disadvantage) to 6 (most severe disadvantage); thus, the total score of the LHS Korean version ranges from 6 to 36, and a higher score indicates greater disability. Cronbach's *α* was 0.791, and the ICC was 0.983, indicating high reliability [18].

#### 2.3.3. Data Analysis

The psychometric properties of the K-USER-P were examined in two parts. First, we assessed test-retest reliability and internal consistency. Second, we had quantitative comparison of the K-USER-P, SF-12, and LHS. Analyses were performed with SPSS 21.0 (SPSS, Chicago, IL). Internal consistency was assessed with the use of Cronbach's alpha, which should be at least 0.70 [19]. Test-retest reliability was assessed with the use of ICC between the first and second measurement. Construct validity was tested by comparing the K-USER-P with the SF-12 and LHS using Spearman's rank correlation test as a nonparametric approach.

## 3. Results

### 3.1. Response and Patient Demographics

A total of 120 questionnaires involving the K-USER-P were distributed to rehabilitation hospitals and centers by mail. We received 100 questionnaires back and included 67 (67%) in the final analysis after excluding questionnaires with insufficient responses. For the test-retest analysis, we also received 20 questionnaires out of a total of 30 and used 13 (65%) for the final analysis. The characteristics for 67 participants were presented in Table 1.

### 3.2. Internal Consistency

Internal consistency of the K-USER-P was determined using Cronbach's alpha. The alpha values were 0.69 for the frequency scale, 0.66 for the restriction scale, and 0.67 for the satisfaction scale, indicating good internal consistency within the assessment (Table 2).

### 3.3. Test-Retest Reliability

Table 3 lists the intraclass correlation coefficient (ICC) for the test-retest reliability. The good reliability was found in the K-USER-P satisfaction scale (ICC = 0.71 (95% CI: 0.28~0.90)). However, the reliability of the K-USER-P frequency (ICC = 0.63 (95% CI: 0.15~0.87)) and K-USER-P restriction scale (ICC = 0.45 (95% CI: −0.11~0.79)) was found to be poor.

### 3.4. Construct Validity

Intercorrelations between the subtests of SF-12 including PCS and MCS and the London Handicap Scale with the K-USER-P scales are listed in Table 4. The K-USER-P scales showed significant, positive correlations with K-SF-12 including PCS and MCS and negative correlations with K-London Handicap. These results indicate that the K-USER-P scales are moderate to good correlated with the K- SF-12 and the K-London Handicap Scales ranging from 0.27 to 0.62.

## 4. Discussion

This study evaluating the reliability and validity of K-USER-P in stroke patients showed that it is a useful instrument for measuring levels of participation in the local community, hospital, and sanatorium settings. The results of this study on internal consistency, test-retest reliability, and construct validity of the K-USER-P were generally moderate to good.

The results of the internal consistency analysis showed moderate reliability in the restriction subscale, frequency, and satisfaction subscale. Our study found that the reliability of the Korean version of the USER-P was not similar to that of the original USER-P, which was studied in multidisciplinary outpatient rehabilitation subject [1]. These results indicated that because the subscales of K-USER-P consisting of activities relate to daily life, social relationship, and work, some subscales such as work, leisure activity, and relationship were not suitable to inpatients among participants of this study. However, the K-USER-P is a useful instrument with moderate reliability. In addition, test-retest reliability was measured as an additional method of assessing reliability. Considering that correlation coefficients in the range of 0.50 to 0.75 generally represent moderate to high reliability and a correlation coefficient of over 0.75 indicates very high reliability [20], this study found high test-retest reliability in the satisfaction and frequency subscales and moderate reliability in the restriction subscales. Of these three areas, the correlation coefficient of the restriction area was somewhat lower, indicating that changes in participation restriction of patients occurred during the 2 weeks after first test administration.

Correlations between the K-USER-P and subscales of SF-12 or the London Handicap Scale were low. The subscales of SF-12 showed a positive correlation with the K-USER-P, and coefficients of correlation were higher in the satisfaction subscale than frequency and restriction subscales. And there is a good correlation between the London Handicap Scale and K-USER-P. Particularly, a correlation satisfaction subscale of K-USER-P is stronger than for the frequency and restriction subscales. Our results suggest that the SF-12 is not sufficiently sensitive or responsive to changes in participation level because the items of the SF-12 are related to health status, while the K-USER-P and the London Handicap Scale measure participation restriction. In addition, the SF-12 contains a small number of items.

One limitation of this study is that only subjects who had experienced stroke were included. Studies involving subjects with other conditions, such as spinal cord injury or musculoskeletal disease, are also needed. Second, because subjects had certain limitations on participation caused by hospitalization, some items of the K-USER-P did not apply. Third, our study lacked methods for assessing the validity of the K-USER-P because we only calculated construct validity. Finally, the number of participants for test-retest analysis is very small. Also, factor analysis was not conducted because of inadequate sample size. However, the K-USER-P showed high reliability similar to that of the original USER-P instrument, indicating that it is a very important tool for identifying activities of daily living participation in Korea. Therefore, our data suggest that the K-USER-P can be reliably used among stroke patients to evaluate the effects of rehabilitation interventions. This study is significant because the K-USER-P can be used by occupational therapists in hospitals and local communities for measurement of the participation level of people with stroke in Korea.

## Figures and Tables

**Table 1:** Patient characteristics (*n* = 67).

Gender (*n*; %)	
Male	46 (68.7)
Female	21 (31.3)
Age in years (mean; SD)	55.3 (13.3)
Education (*n*; %)	
No education	10 (14.9)
Elementary school	5 (7.5)
Middle school	6 (9.0)
High school	30 (44.8)
College/university	16 (23.8)
Occupation (*n*; %)	
Yes	11 (16.4)
No	56 (83.6)
Residence (*n*; %)	
Own house	17 (25.4)
Rehabilitation hospital	43 (64.2)
Care hospital	7 (10.4)
Marital status (*n*; %)	
Married	36 (53.7)
Unmarried	17 (25.4)
Divorced	3 (4.5)
Widowed	11 (16.4)
Caregiver (*n*; %)	
None	25 (37.3)
Family	13 (19.4)
Caregiver	29 (43.3)

**Table 2:** Internal consistency of K-USER-P (*N* = 67).

K-USER-P frequency	K-USER-P restriction	K-USER-P satisfaction
0.69	0.66	0.67

Note: K-USER-P: Korean version of Utrecht Scale for Evaluation of Rehabilitation-Participation; data represent Cronbach's *α*.

**Table 3:** Test-retest reliability (*N* = 13).

Items	Test	Retest	Mean difference	*p*	ICC	95% CI
	M (SD)	M (SD)	M (SD)			
K-USER-P frequency	15.71 (12.10)	12.53 (10.15)	3.19 (9.58)	0.25	0.63	0.15~0.87
K-USER-P restrictions	40.79 (23.66)	42.2 (28.6)	−1.40 (27.54)	0.86	0.45	−0.11~0.79
K-USER-P satisfaction	35.19 (18.91)	35.39 (21.29)	−0.19 (15.36)	0.97	0.71	0.28~0.90

**Table 4:** Construct validity for USER-P (*N* = 67).

K-USER-P	K-SF-12			K-London Handicap
	PCS	MCS	Total	
	Rho	Rho	Rho	Rho
K-USER-P frequency	0.33^*∗∗*^	0.35^*∗∗*^	0.38^*∗∗*^	−0.54^*∗∗*^
K-USER-P restriction	0.41^*∗∗*^	0.27^*∗*^	0.35^*∗∗*^	−0.58^*∗∗*^
K-USER-P satisfaction	0.43^*∗∗*^	0.45^*∗∗*^	0.50^*∗∗*^	−0.62^*∗∗*^

Note: K-USER-P: Korean version of Utrecht Scale for Evaluation of Rehabilitation-Participation; K-SF-12: The 12-Item Short-Form Health Survey (SF-12) Korean version; K-London handicap: London Handicap Scale Korean version.

Note: data represent Pearson's correlation.

^*∗*^ *p* value  < 0.05 (bilateral).

^*∗∗*^ *p* value <  0.01 (bilateral).

## References

[1] Post M. W. M., van der Zee C. H., Hennink J., Schafrat C. G., Visser-Meily J. M. A., van Berlekom S. B. (2012). Validity of the utrecht scale for evaluation of rehabilitation-participation. *Disability and Rehabilitation*.

[2] World Health Organization (2011). *International Classification of Functioning, Disability and Health: ICF*.

[3] Park E.-Y., Choi Y.-I. (2014). Rasch analysis of the London Handicap Scale in stroke patients: a cross-sectional study. *Journal of NeuroEngineering and Rehabilitation*.

[4] Timmermans A. A., Seelen H. A., Willmann R. D., Kingma H. (2009). Technology-assisted training of arm-hand skills in stroke: concepts on reacquisition of motor control and therapist guidelines for rehabilitation technology design. *Journal of NeuroEngineering and Rehabilitation*.

[5] Hammel J., Magasi S., Heinemann A., Whiteneck G., Bogner J., Rodriguez E. (2008). What does participation mean? An insider perspective from people with disabilities. *Disability and Rehabilitation*.

[6] Magasi S., Post M. W. (2010). A comparative review of contemporary participation measures' psychometric properties and content coverage. *Archives of Physical Medicine and Rehabilitation*.

[7] Post M. W. M., de Witte L. P., Reichrath E., Verdonschot M. M., Wijlhuizen G. J., Perenboom R. J. M. (2008). Development and validation of IMPACT-S, an ICF-based questionnaire to measure activities and participation. *Journal of Rehabilitation Medicine*.

[8] Brown M., Dijkers M. P. J. M., Gordon W. A., Ashman T., Charatz H., Cheng Z. (2004). Participation objective, participation subjective: a measure of participation combining outsider and insider perspectives. *Journal of Head Trauma Rehabilitation*.

[9] Van Der Zee C. H., Priesterbach A. R., Van Dussen L. D. (2010). Reproducibility of three self-report participation measures: the ICF measure of participation and activities screener, the participation scale, and the utrecht scale for evaluation of rehabilitation-participation. *Journal of Rehabilitation Medicine*.

[10] Hong K.-S., Bang O. Y., Kang D.-W. (2013). Stroke statistics in Korea: part I. Epidemiology and risk factors: a report from the Korean stroke society and clinical research center for stroke. *Journal of Stroke*.

[11] Tse T., Douglas J., Lentin P., Carey L. (2013). Measuring participation after stroke: a review of frequently used tools. *Archives of Physical Medicine and Rehabilitation*.

[12] Hartman-Maeir A., Soroker N., Ring H., Avni N., Katz N. (2007). Activities, participation and satisfaction one-year post stroke. *Disability and Rehabilitation*.

[13] Obembe A. O., Eng J. J. (2015). Rehabilitation interventions for improving social participation after stroke: a systematic review and meta-analysis. *Neurorehabilitation and Neural Repair*.

[14] Van Der Zee C. H., Kap A., Mishre R. R., Schouten E. J., Post M. W. M. (2011). Responsiveness of four participation measures to changes during and after outpatient rehabilitation. *Journal of Rehabilitation Medicine*.

[15] Streiner D. L., Norman G. R. (2008). *Health Measurement Scales: A Practical Guide to Their Development and Use*.

[16] Yoo S. H. (2006). *The Characteristics and Risk Factors of Pain Among Older Adults in Single District of Korea*.

[17] Lim L. L.-Y., Fisher J. D. (1999). Use of the 12-item Short-Form (SF-12) Health Survey in an Australian heart and stroke population. *Quality of Life Research*.

[18] Choi Y. I., Kim W. H., Park E. Y. (2011). Validity and reliability of the Korean version of the London handicap scale. *Journal of the Korea Academia-Industrial Cooperation Society*.

[19] Terwee C. B., Bot S. D. M., de Boer M. R. (2007). Quality criteria were proposed for measurement properties of health status questionnaires. *Journal of Clinical Epidemiology*.

[20] Portney L. G., Watkins M. P. (2009). *Foundations of Clinical Research: Applications to Practice*.

